# Wearables for Engagement Detection in Learning Environments: A Review

**DOI:** 10.3390/bios12070509

**Published:** 2022-07-11

**Authors:** Maritza Bustos-López, Nicandro Cruz-Ramírez, Alejandro Guerra-Hernández, Laura Nely Sánchez-Morales, Nancy Aracely Cruz-Ramos, Giner Alor-Hernández

**Affiliations:** 1 Instituto de Investigaciones en Inteligencia Artificial, Universidad Veracruzana, Xalapa, Veracruz 91097, Mexico; maritbustos@gmail.com (M.B.-L.); ncruz@uv.mx (N.C.-R.); aguerra@uv.mx (A.G.-H.); 2 Division of Research and Postgraduate Studies, CONACYT-Tecnológico Nacional de México/I. T. Orizaba, Av. Oriente 9 852 Col. Emiliano Zapata, Orizaba, Veracruz 94320, Mexico; laura.sm@orizaba.tecnm.mx; 3 Division of Research and Postgraduate Studies, Tecnológico Nacional de México/I. T. Orizaba, Av. Oriente 9 852 Col. Emiliano Zapata, Orizaba, Veracruz 94320, Mexico; dci.ncruz@ito-depi.edu.mx

**Keywords:** engagement detection, learning environments, physiological signals, sensors, wearables

## Abstract

Appropriate teaching–learning strategies lead to student engagement during learning activities. Scientific progress and modern technology have made it possible to measure engagement in educational settings by reading and analyzing student physiological signals through sensors attached to wearables. This work is a review of current student engagement detection initiatives in the educational domain. The review highlights existing commercial and non-commercial wearables for student engagement monitoring and identifies key physiological signals involved in engagement detection. Our findings reveal that common physiological signals used to measure student engagement include heart rate, skin temperature, respiratory rate, oxygen saturation, blood pressure, and electrocardiogram (ECG) data. Similarly, stress and surprise are key features of student engagement.

## 1. Introduction

Student engagement in the classroom is usually directly linked to the student’s perception of the pedagogical activities and strategies implemented in class. Student engagement is usually measured when teaching reading comprehension. In many cases, reading is a fundamental building block to students’ development and success both in and out of the classroom. It strengthens the brain and promotes critical thinking. Similarly, reading comprehension as a skill allows students to interpret written discourse. However, teaching reading comprehension may be a challenging task when it comes to keeping students fully engaged.

In their work, Bosch et al. [1] identified three types of student engagement: affective, behavioral, and cognitive. Current methods for monitoring engagement levels among students during educational activities usually rely on computer vision for image processing and recognition of facial expressions, gestures, postures, and eye movements. Similarly, physiological and neurological sensors attached to wearables can capture key physiological features as indicators of student engagement.

The computer vision approach allows researchers to extract valuable data on the affective, cognitive, and behavioral states of students during specific learning activities and from three channels: audio, image, and video. The data obtained from computer vision allow experts to monitor and measure to what extent students remain engaged in a particular classroom activity, initially designed to teach something. In parallel, the use of wearables in learning environments allows for detecting data of factors involved during learning experiences, such as concentration, engagement, and attitude to name but a few, through the monitoring of physiological signals. Such data is key for teachers, as it allows them to understand why students perform in a certain way in class.

A substantial amount of scientific literature includes reviews or proposes sensors and biosensors to measure physiological variables. Castaneda et al. [2], Shabaan et al. [3], Lou et al. [4], Guo et al. [5] and Tandon et al. [6] developed their own sensors and wearable technologies for monitoring of different physiological signals. Nahavandi et al. [7] analyzed the challenges and opportunities of Artificial Intelligence (AI)-based wearable devices. In turn, researchers Reda et al. [8], Surantha et al. [9], Khoshmanesh et al. [10], Santo et al. [11], Akinosun et al. [12], DeVore et al. [13] and Burnham et al. [14] have introduced their own reviews of wearables applied to healthcare. In this perspective, some parameters used in these works are relevant for engagement in students, such as heart rate, blood pressure, postures, sleep, to mention but a few. Other researchers [15,16,17,18,19] have proposed works focuses on sensors and wearables for physiological and behavioral monitoring of students, eye-tracking devices for capturing student attention and understanding Engagement in Learning.

Emotional state and posture recognition have become an important topics for student engagement detection [20,21,22,23,24,25]. Additionally, contributions such as that of Salmeron-Majadas et al. [26] seek to collect and process keyboard and mouse interactions to measure how students perform during learning activities. Other works centered around student emotional state detection analyze and process signals from Electroencephalogram (EEG), Electromyogram (EMG), Electrocardiography (ECG), Electrodermal activity (EDA), heart rate variability, skin temperature, blood volume pulse, respiration, or Electrodermography (EDG)/galvanic skin response (GSR) [27,28,29,30,31,32,33,34,35,36,37]. Researchers [38,39,40,41,42,43,44,45,46,47] report the use of deep learning and machine learning (ML) techniques for emotion classification. Finally, other techniques rely on emotion recognition via computer vision [22,41,48,49,50], linguistic semantic approaches [51], and biological features [52].

Our analysis of the aforementioned works leads us to conclude that emotion recognition based on physiological signals is highly applicable to the study of student learning processes. Most of the physiological signals involved in such studies analyze and process EEG data from wearable devices such as wristbands or headbands. Other studies rely on strategies that combine facial expression recognition with the monitoring of vital signs and other factors, such as keystrokes, body movements, muscle pressure, or gesture rigidity. It also seems that Machine Learning Algorithms (MLA) and computer vision are the most common techniques to detect student emotion states and engagement through factors such as facial expressions, eye movement, and speech. Some deep-learning-based algorithms have been developed to monitor in real time emotions such as anger, disgust, fear, happiness, sadness, and surprise. These algorithms compute Mean Engagement Score (MES) by analyzing data retrieved from facial landmark detection and emotional recognition.

From this understanding, the use of information technologies for engagement level detection in different educational subjects such as Spanish, history, science, or mathematics based on innovative technologies such as the recognition of physiological signals represents an opportunity to improve the teaching–learning process.

This research has four objectives that distinguish it from similar reviews. First, we review and identify commercial and non-commercial wearables that use sensors for engagement detection. Second, we identify the common sensors attached to those wearables. We also highlight the key physiological variables involved in student engagement detection and monitoring. Finally, we review and discuss the FDA approval status of the reviewed commercial wearables.

## 2. Main Physiological Signals for Student Engagement Detection

**Figure 1:**
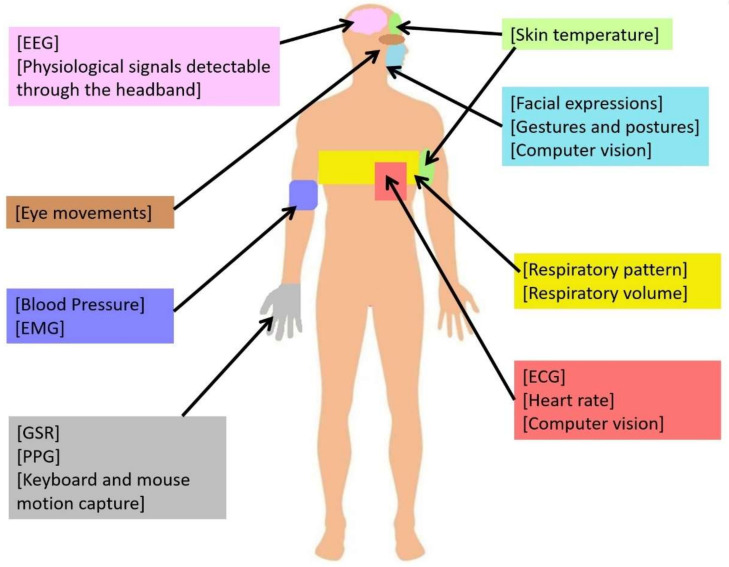
Physiological Variables for Engagement Detection and Associated Body Parts.

The sources of data for the collection of emotional and physiological data in learning environments are diverse. According to Feidakis [53], some tools can compute physiological signal readings, whereas some others allow for observing behavioral activity. Some parameters and signals commonly used for emotion state detection and physiological monitoring are introduced below [53]. Additionally, Figure 1 visually illustrates the body parts commonly associated to the measurement of these parameters and signals:

*Electroencephalography (EEG)*: According to Rogers [54], EEG is a technique for registering and analyzing the brain’s electrical activity. Neurons, also referred to as brain nerve cells, produce electrical impulses that oscillate rhythmically in different patterns. An EEG is used to measure and record brain activity patterns. The instrument’s recording is called an electroencephalogram. In educational contexts, EEG measurements should provide a more objective indication of how the brain functions during learning activities over time in comparison to the think-aloud type of self-reporting technique. Additionally, EEG can distinguish between the different brain’s active states quantitatively by analyzing the wavelength band.

*Electrocardiogram (ECG)*: An ECG records the natural electrical impulses that coordinate the contractions of the various parts of the heart to show the rate at which the heart beats, the rhythm of the beats (steady or irregular), and the strength and timing of the electrical impulses as they travel through the various parts of the heart [55]. In a learning environment, monitoring ECG patterns can help track students’ attention in the classroom and their cognitive activities.

*Blood Pressure (BP)*: BP refers to the force of the blood pushing against the walls of the arteries. It is measured in millimeters of mercury, and it is expressed as a measurement with two numbers, one number on top (systolic), and the other one on the bottom (diastolic), as in a fraction (for instance, 120/80 mmHg). Systolic pressure is the pressure of the blood when the heart beats or contracts. Conversely, diastolic pressure is the pressure of the blood between beats; that is, when the heart relaxes [56]. According to Taj-Eldin et al. [57], measuring BP helps monitor changes in a person’s emotional state, such as stress. If a student experiences a stressful situation, the body produces hormones that temporarily increase BP.

*Electromyogram (EMG)*: An EMG is a test that measures electrical discharges from muscles. The test is performed by placing a thin needle into a muscle and measuring its electrical activity at rest and during use [58]. Monitoring EMG signals can help identify student emotional arousal during educational activities. Measuring the movement of facial muscles through EMG provides parameters to study student behavior during interactions with dynamic visual content.

*Skin temperature (ST)*: According to Yasuma and Hayano [59], skin temperature is measured on the surface of the human skin; thus, only skin contact is required. Furthermore, including body and peripheral temperature as a physiological parameter is useful for detecting emotions such as stress. The use of skin sensors to monitor parameters such as skin temperature facilitates emotion detection and allows researchers to measure student engagement in different educational activities, such as watching movies and role playing [60].

*Galvanic Skin Response (GSR)*: It measures skin conductance. GSR is measured by placing two electrodes on the skin surface, namely on the fingers. One electrode applies a small amplitude alternating current into the skin, and the other is used to calculate skin impedance using Ohm’s Law given a voltage [61]. GSR is a function of skin moisture level that is related to the sweat glands. Through the sweat glands it is possible to measure emotional arousal, which leads to an increase in sweat gland activity. In the educational context, research has measured both student engagement and emotional arousal during educational activities such as reading using GSR data [62].

*Photoplethysmography (PPG)*: According to Allen [63], PPG is a simple and affordable optical technique used to detect changes in blood volume in the microvascular bed of tissues. It is often used noninvasively for measurements on the skin surface. In educational research, PPG sensors have been useful for measuring parameters such as heart rate to detect student cognitive engagement during learning activities.

*Respiratory pattern (RP), Respiratory volume (RV) and heart rate (HR)*: According to Braun [64], in the ventilation process that allows the movement of air into the lungs, the respiratory system has a central respiratory pacemaker within the medulla of the brainstem. Neuronal output travels from this center by the spinal cord to the respiratory muscles. The changes made by the inspiratory and expiratory muscles, as they contract and relax, cause a rhythmic breathing rate and pattern. Changes in respiratory patterns are related to positive emotions such as happiness. Some of the changes include increased variability in the respiratory pattern or decreased respiratory time. On the other hand, respiratory volume relates to the volume of gas present in the lungs at a specific time during the respiratory cycle [65]. Some lung volume parameters, such as inspiratory reserve volume, tidal volume, and expiratory reserve volume, are measured by spirometry. However, functional residual capacity, total lung capacity, and residual volume are measured by body plethysmography, nitrogen washout, and helium dilution. Finally, heart rate refers to the number of times the heart beats per minute and is used to monitor cardiac activity [57]. Even though it provides partial information on the heart’s activity, monitoring heart rate is useful for measuring student engagement in different teaching modalities. For instance, during active learning activities based both on problem-solving and peer discussion, heart rate tends to increase [66].

*Facial Expression Recognition (FER)*: Facial expressions are changes that occur in the human face [67]. According to Dewan et al. [41], facial expressions are directly associated with perceived engagement. One of the methods for the detection of facial expressions is the analysis of facial images. Analysis methods used to extract information about engagement use geometric and holistic features. Such methods are classified in part-based and appearance-based methods. The study of FER among students is useful to identify their emotions, which helps teacher recognize whether students understand or not a given topic, for example.

*Gestures and postures*: According to Dewan et al. [41], gestures and posture are forms of nonverbal communication expressed by human body language. They are linked to emotional-cognitive states that either favor or hinder learning. In their research, Dewan et al. [41] collected data from webcam video recordings, skin conductance, and Kinect depth video to infer student engagement. The analysis of gestures such as hand movements helps determine a person’s intention when performing an action, which allows teachers and experts to detect student attention or disengagement in the learning process.

*Eye movements*: Eye movements have been widely used to understand the emotional states of students during online educational activities [41]. In their work, Dewan et al. [41] review a series of studies in which eye movement patterns, head movements, and facial features measure the level of student engagement and concentration in online learning environments.

*Keyboard and mouse motion capture*: The mouse motion technique analyzes features such as average speed, inactivity, the orientation of mouse movements, mouse speed, acceleration, hand agitation, click coordinates, scrolling, temperature, humidity, and user keystroke intensity to detect a person’s mood. On the other hand, analyzing keyboard movements to determine student affective states or engagement implies analyzing elements such as keystroke verbosity (number of keys and blanks), keystroke time (latency measures), pause behaviors, typing speed, the number of characters typed during a 5-s interval, total typing time, and idle times [26]. Overall, the analysis of mouse and keyboard interaction patterns during tasks such as free text typing enables the study of student affective states during learning activities.

*Physiological signals detectable through the headband*: Most of the physiological signals detected through a headband use EEG with features such as Power Spectral Density (PSD), Signal Power (SP), and Common Spatial Pattern (CSP) [27]. Signals measured through a headband can help monitor student concentration during educational activities, which is in turn helpful for teachers to monitor student engagement in class.

## 3. Methods

This paper is a review of sensor technologies from the IoT perspective. We highlight how sensors attached to wearables manage to detect and monitor student cognitive engagement in learning environments through the recording and processing of physiological signals. We rely on the PRISMA [68] statement to ensure research clarity and methodological robustness.

**Inclusion and exclusion criteria**. Initially, we retrieved 4142 queries from a selected set of databases. Below, we describe the review inclusion and exclusion criteria:

*Inclusion criteria*. We searched for studies published from 2010 to 2021 on (1) engagement detection in learning environments, (2) physiological signal monitoring, (3) commercial and non-commercial wearable devices and sensors, (4) IoT wearable devices, and (5) FDA-approved medical devices.

*Exclusion criteria*. We discarded search results of researches that (1) were not written in English, (2) were presented in the form of reports and letters, (3) was not a first study, and (4) were not peer-reviewed.

**Figure 2:**
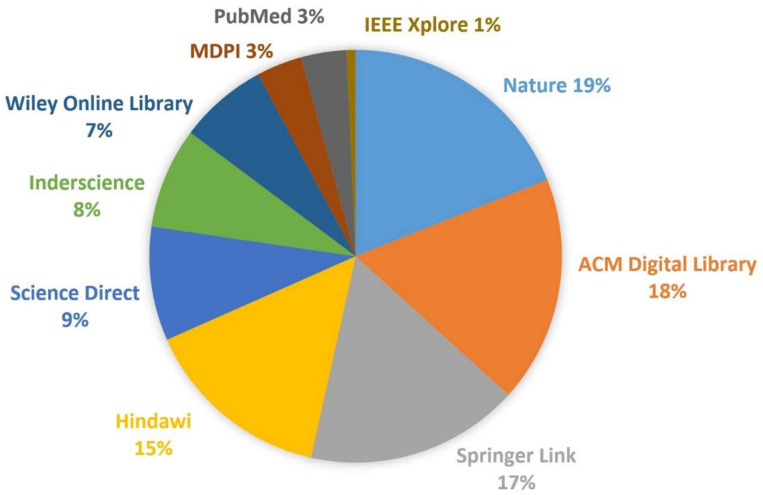
Distribution of Information Sources.

**Information Sources**. The keywords of our research questions were identified and classified into two groups: computing technology and engagement detection. These areas of knowledge helped us to determine the specialty of the scientific digital library chosen as an information source. In terms of engagement detection, we relied on PubMed (3%) and Nature (19%), whereas in terms of computing technology, we chose Science Direct (9%), Wiley Online Library (7%), IEEE Xplore (1%), Hindawi (15%), Inderscience (8%), Springer Link (17%), ACM Digital Library (18%), and MDPI (3%). During federate search on Google Scholar, all these scientific libraries yielded good results. Once the libraries were selected, we retrieved relevant studies by submitting search queries to the corresponding search engines of each digital library (See Figure 2). The search was performed from January to December 2021.

**Search Strategy**. We combined keywords using Boolean-like connectives to filter the search results. The keywords of the search were extracted from the keywords that shaped the research questions. The search strategy resulted of a series of intermediate searches that led to the answer to the research question. These intermediate searches were ordered to determine the search terms to be used in the posterior queries:

1. Sensors and biosensors that measure these physiological variables;
2. FDA-approved commercial wearable devices for engagement detection in learning environments;
3. Commercially available consumer wearable devices using sensors for engagement detection in learning environments;
4. Non-commercial wearable devices using sensors for engagement detection in learning environments;
5. Physiological variables involved in engagement detection in learning environments.

Queries 3 and 4 were used to the databases. Query 3 resulted in the following search expression, which used adjacent search terms conjugated with AND and OR connectives as follows:

‘Engagement Detection’ AND ‘Learning Environments’ AND (‘Physiological Signals’ OR ‘Physiological Variables’ OR ‘Physiological Parameters’) AND (‘students’ OR ‘young people’) AND ‘wearable’.

Results showed that the physiological variables involved in the students’ emotions were the relevant search terms. Query 4 integrated these search terms into a search expression whose implementation produced new results associated with the physiological variables. Similarly, as the results of each of the queries detailed above generated new search terms, the results were progressively expanded to those that were relevant to this study. The results of the last phase of consultation included those wearable devices that contain sensors (biosensors) which measure physiological variables in students. The wearable devices included could be commercial or non-commercial and, in the first case, they could be FDA-approved or not.

**Figure 3:**
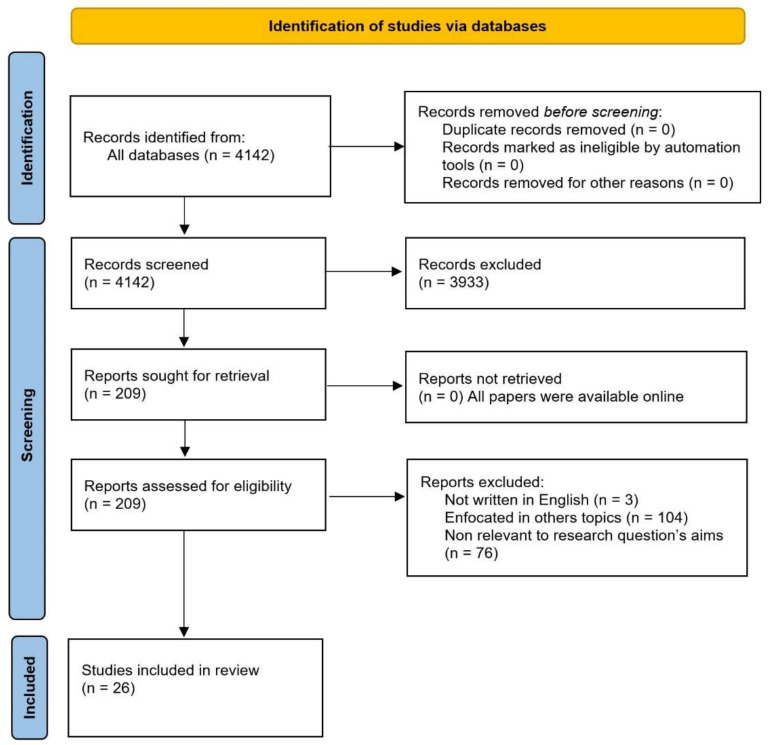
PRISMA Flow Diagram of the Search Strategy.

**Selection process**. Three subject matter experts (SMEs) screened the abstracts and titles of the 4142 relevant papers retrieved at the search stage. Next, the information was grouped into eight categories: wearable manufacturer, model, form factor (it refers to the size, type, and physical specifications of the device), sensors used, measured parameters, physiological signs detected, API, and FDA status. Following this first analysis 3933 papers were discarded. The remaining 209 papers became of interest for a more detailed analysis of their content. Following this second analysis, we excluded 183 more papers. Finally, only the remaining 26 papers were selected for this review. These papers came from the following digital libraries: PubMed (3), Nature (5), IEEE Xplore (2), ScienceDirect (1), MDPI (2), Springer Link (2), ACM Digital Library (4), and other sources (6). In Figure 3, the PRISMA diagram details our study search and selection strategy.

**Data collection and analysis**. We categorized relevant information from the studies and migrated it to structured tables for a thorough analysis. The database thus contained details on current commercial and non-commercial wearables and sensors for engagement detection. We did not perform a randomized controlled selection, since we found a relatively small number of wearables; only 26. Information of interest for each wearable was as follows: device manufacturer, model, form factor, sensors used, parameters measured, physiological signs being detected, API, and FDA approval status.

## 4. Results

### 4.1. Study Selection

We initially collected 4172 records across the digital libraries mentioned in the beginning of this section and listed in Figure 3. These records were then screened to assess their relevance, thus discarding 3933 of them. The eligibility of the remaining 209 records were evaluated by analyzing the relevance of their full-text content. The evaluation excluded 183 records for several reasons, including the following: (i) the papers were not writ ten in English, (ii) they focused on topics irrelevant to the research, and (iii) they were irrelevant to the aim of the research questions. Therefore, only 26 studies were included in the review as primary researches after applying the inclusion and exclusion criteria for eligibility.

### 4.2. Study Characteristics

We grouped the identified wearable devices into two large categories: commercial wearables and non-commercial wearables. Commercial wearables included those being manufactured, those already found on the market, and those being in presale by the time of writing this paper. Conversely, non-commercial wearables comprised all those wearables still found at prototype phase and those reported in scientific literature but not yet being manufactured by the time of writing this paper. Reviews of each device category, including 79 wearable devices and 15 non-commercial wearable devices, are presented below.

#### 4.2.1. Classification of Wearables for Learning Engagement Detection in Learning Environments

In terms of the biosensors comprised in the wearables, Table 1 presents our classification. This classification is based on the analyses of wearable biosensor technologies proposed by [69,70], and it groups our findings into three categories: mechanical biosensors, physiological biosensors, and biochemical biosensors. The table also lists the technologies upon which these sensors rely, their applications in engagement detection, and the types of wearables using these sensors.

#### 4.2.2. Commercial Wearables for Engagement Detection

Our review of commercial wearables for engagement detection comprises commercially available wearables that set monitoring metrics for detecting engagement. However, notice that most of these devices do not limit themselves to engagement detection in learning environments, as they are reported to have other applications. Our findings revealed that a wide range of commercial wearable devices for monitoring physiological parameters and detecting emotional states have applications in healthcare [70,71]. Table 2 presents our classification of commercial wearable devices for engagement detection. This classification takes into account the following aspects of each device: manufacturer, model, form factor, parameters measured, physiological signs detected, and APIs. Our most important findings concern the different wearable form factors commercially available, including armbands, chest belts, wrist monitors, chest patches, chest straps, contactless in-bed sensors, earbuds, headbands, smart rings, smartwatches, wristbands, and T-shirts. Both smartwatches and wristbands are the most common form factors among manufacturers. Examples of smartwatches include the Huawei Watch 3, Venu^®^ Sq from Garmin, the Apple Watch Series 7, and the Samsung Gear Sport. As regards the parameters measured by these wearables, the following stand out: skin temperature, oxygen saturation, respiratory rate, heart rate, heart rate variability, blood pressure, EEG, stress levels, sleep, EMG, step tracking, and PPG. On the other hand, the most common physiological signals involved in engagement detection are stress, relaxation, surprise, postures, engagement, concentration, and laugh. Finally, each wearable relies on different type of software, which depends on the physical components of each device and the parameters that can be measured.

We can identify the contribution of the devices to assist and increase student engagement during educational activities. We found that the Chest strap, Smartwatch and Leap Motion can detect ECG signals, and their application is focused on gait tracking and motion tracking. Meanwhile, the Armband, Wristband, Ring, Abdominal patch, Headband and GSR Velcro electrodes can detect PPG and GSR signals, which have application in concentration monitoring. Furthermore, epidermal patches and textile patches can detect ECG signals in an inhaled form that facilitate the detection of movement in the extremities.

Additionally, ECG signals such as heart rate or heart rate variability are associated with stress, surprise, relaxation and concentration. Meanwhile, PPG signals such as blood volume or oxygen saturation are related to stress, laughter, interest and frustration. In both cases, physiological signals allow the detection of students’ engagement in educational activities.

**Figure 4:**
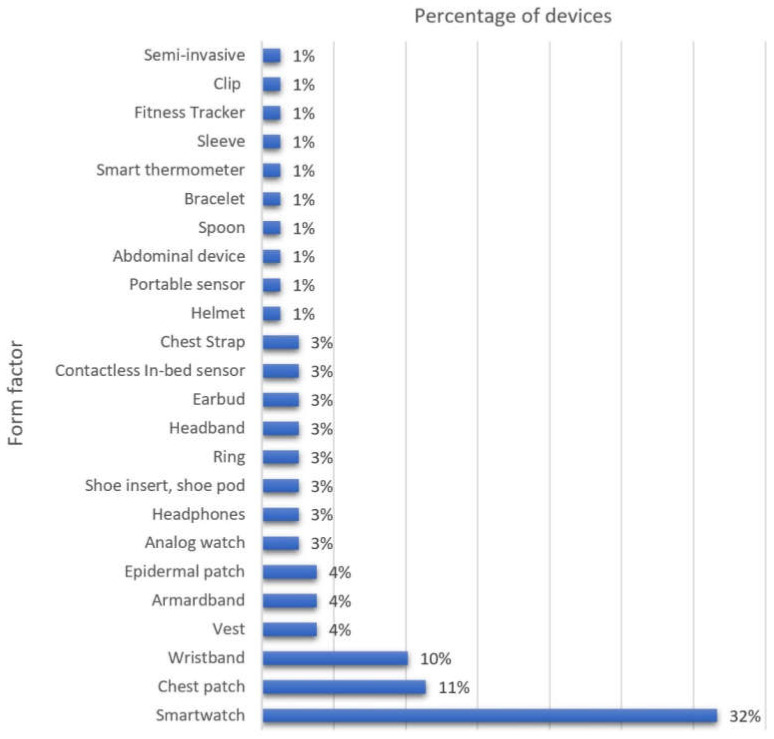
Incidence of Commercial Wearable Form Factors in the Literature.

Most of the commercial wearables can record important physiological characteristics, such as respiratory rate, barometric pressure, posture, skin temperature, muscle movement, blood oxygenation, and heart rate to name but a few. Further, such devices usually transfer the recorded data to be processed using wireless technology. Once the data is processed, wearable users can visualize the data reports via a mobile application. As our findings revealed, commercial wearable devices are mostly smartwatches (32%). Conversely, less commonly manufactured wearables include helmets, bracelets, spoons, smart thermometers, smart sleeves, and fitness trackers (1%). Devices with 10–11% of incidence in the review include wristbands and chest patches, whereas headphones, analog watches, headbands, smart rings, earbuds, contactless in-bed sensors, and chest straps only showed 3% of incidence. Figure 4 introduces a graph for the classification of the commercial wearable form factors reported in the literature.

**Figure 5:**
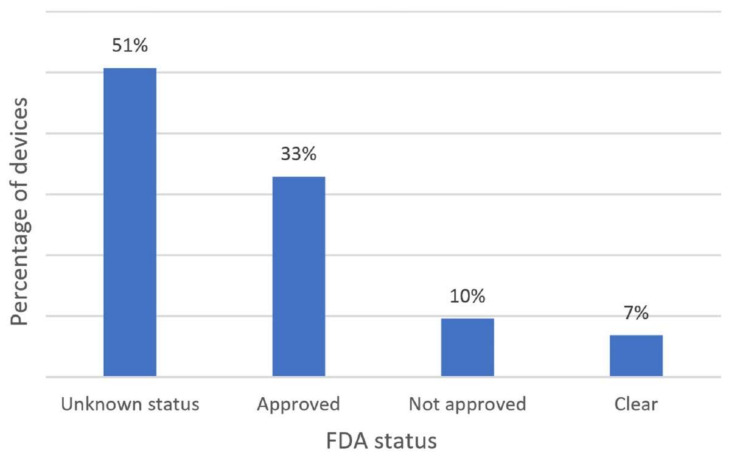
FDA Approval Status of Commercial Wearables for Engagement Detection.

Wearable devices with healthcare applications must be approved in terms of reliability and efficiency. FDA-approved wearables for engagement detection have proved to be efficient in measuring physiological signals for detecting and monitoring engagement. Each wearable reported in the literature holds one of the four FDA statuses: approved, clear, unknown, and unapproved. Our findings indicated that 51% of the devices reported in the literature had an unknow FDA status, which is mainly due to the fact that most device manufacturers do not disclose such type of information. Conversely, 33% of the devices have a public FDA registration. This information is summarized and visually presented in Figure 5.

As regards the physiological signals involved in engagement detection, our findings indicate that most of the commercial wearables can detect more than physiological signal simultaneously, especially surprise, stress, interest, relaxation, and laugh. Table 3 summarizes our findings of the commercial wearables reported in the literature with respect to both the physical signs being monitored and the FDA approval status of each device.

**Figure 6:**
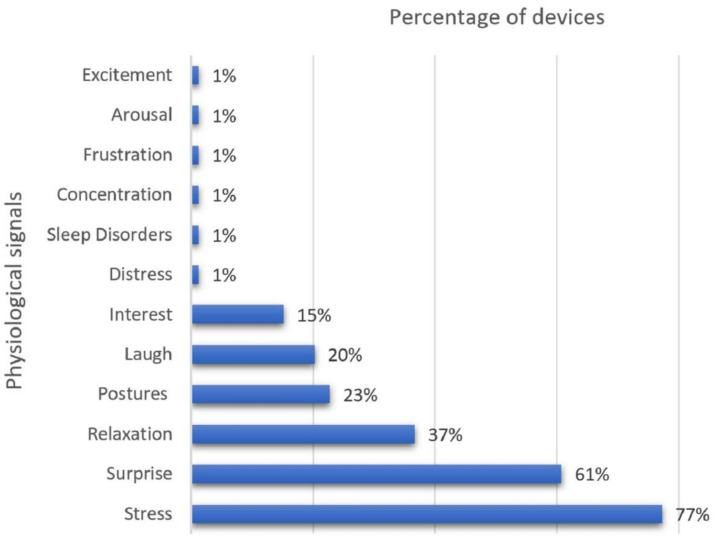
Correspondence between Commercial Wearables and Physiological Signals for Engagement Detection.

Figure 6 graphically illustrates the distribution of the commercial wearables reported in the literature with respect to the physiological signals for engagement detection.

As our findings indicate, several commercial wearables reported in the literature can detect more than one physiological signal simultaneously, especially surprise, stress, interest, relaxation, and laugh, through monitoring techniques for respiratory rate, oxygen saturation, blood pressure, and heart rate.

#### 4.2.3. Non-Commercial Wearables for Engagement Level Detection

Non-commercial wearables for engagement detection are mostly developed for research purposes or are still at the development phase of the manufacturing cycle. Table 4 summarizes our review of non-commercial devices for emotion and engagement detection. The table highlights the key characteristics of each wearable, which are also listed below:

- Aim: Physiological signal(s) monitored by the wearable.
- Device type: Form factor of the device (e.g., smartwatch, bracelet, headband).
- Function: Brief description of the device’s functionality.
- Sensors: Sensor technologies used to record physiological signal data.
- Real-time monitoring capability: Whether the device can monitor physiological signals in real time.
- Educational environment: Type of educational environment where the wearable has been implemented.

**Table 4:** Non-Commercial Wearables for Engagement Detection.

Aim	Device Type	Function	Sensors	Real-Time Monitoring Capabilities	Educational Environment
Physical exertion, health, and heart function monitoring; tracking an individual’s performance and exertion level [118].	Patch	It consists of a patch that includes a sensor to measure a biochemical (lactate) and an electrophysiological (electrocardiogram) signal to monitor physical exertion, health, and heart. The patch can recognize emotions such as stress and anger.	Lactate sensor, electrophysiological sensors	Yes	Unstated
Skin temperature measuring [119].	Patch	The patch detects multimodal biosignals, measures skin temperature with a sensitivity of 0.31 Ω/°C, skin conductance with a sensitivity of 0.28 μV/0.02 μS, and pulse wave with a response time of 70 msec.	ST, skin conductance, and pulse wave sensors	Yes	Unstated
Heart rate monitoring [120].	Scarf	It helps users to reflect on their emotional state, modify their affective state, and interpret the emotional states of other people. The design of SWARM is based on a scarf so that people with different disabilities have access to this type of technology. SWARM can detect emotions such as stress, sadness, calm, happiness, and excitement.	Biosensors	Yes	Unstated
Heart rate and skin conductance monitoring [121].	Scarf	It is a design of a wearable device based on a scarf form factor. The device features color-changing and olfactory properties to affect people’s emotional state. The wearable comprises two sensors: a heart rate sensor and a skin conductance sensor. When changing color and emitting an odor, the scarf potentiates positive emotions and reduces negative ones.	HR and EDA sensors	Yes	Unstated
Blood volume pulses and muscle contraction monitoring [122].	Glove	It is an emotion recognition framework using machine learning of physiological patterns. The framework relies on a PPG sensor for heart rate monitoring, an EDA sensor, a skin temperature sensor, and an EMG sensor. The proposal focuses on the preprocessing of emotion recognition and supports the recognition of emotions such as happiness, anger, fear, disgust, and sadness.	PPG and EMG sensors	Yes	Unstated
Physiological arousal detection and monitoring [123].	Gloves, bracelet	The device monitors the student’s psychological and physical condition using heart rate, skin conductivity, and respiration sensors. The data obtained are sent to an assistive host to process, analyze, and evaluate student moods and stress levels.	HR sensor, EDA sensor, respiratory rate sensor	Yes	Mobile
Detection of eye movements, eyes closed, and teeth clenching [124].	Eyeglasses	AttentivU is a device that monitors physiological data to measure the engagement and enhance learning activities using silver electrodes. The data collected by the device can be processed in real time or sent to a separate computer.	EEG sensor or electrooculography (EOG)	Yes	Unstated
Monitoring of physiological characteristics related to heart rate, oximetry, skin temperature, and GSR [125].	Patch	The proposal uses an Arduino board to obtain physiological signals from the user and connected sensors to acquire data on skin temperature, GSR, pulsometer, and a respiratory rate sensor. The data is processed using Matlab.	ST sensor, oximeter breath-flow rate sensor, HR sensor, GSR sensor	Yes	Unstated
Heart rate monitoring [126].	Shirt	The prototype is based on an Arduino Uno board to which is connected a pulse sensor that uses infrared light to detect user heart rate.	Pulse sensor	Yes	Unstated
Human physical activity monitoring [127].	Shirt	SensVest is a wearable prototype to monitor physical aspects. The device includes a series of sensors that allow the recording of different data related to human performance to improve the understanding of scientific concepts in students.	HR sensor, ST sensor, accelerometer	Yes	Unstated
Heart rate and breathing rate monitoring [128].	Patch	The device obtains an ECG tracing using two electrodes in symmetrical positions on the user’s body and a third ground electrode placed next to one of the sensing electrodes.	Electrodes, ECG sensor	Yes	Computer video
Heart rate variability monitoring, skin temperature measuring [129].	Wristband	n-Gage is a system that evaluates the engagement levels of behavioral, emotional, and cognitive students. The system detects the student’s physical and physiological signals and environmental changes in the educational environment.	EDA sensor, accelerometer, ST sensor	Unstated	Unstated
EDA and pulse rate monitoring [130].	Patch	The proposal measured and recorded electrodermal activity, pulse rate, and facial recognition during an e-learning session to determine the level of student engagement. The data collected were analyzed using software developed with Matlab.	EDA sensor, HR sensor	Yes	E-learning
Upper body pressure distribution [131].	Chair	While students perform e-learning reading activities, the student’s upper body pressure is recorded using a chair with a pressure mat. The data is processed using classifiers.	Pressure mat	No	E-learning
Feet posture and movement detection [132].	Insole	This platform contains an insole with ground contact force (GCF) plantar pressure sensors. In addition, a microcontroller with WIFI technology collects the data and sends it to a database to be analyzed by a Human Activity Recognition classifier.	Accelerometer, gyroscope sensor, magnetometer, barometer, and range finder sensors	Yes	No specified

**Figure 7:**
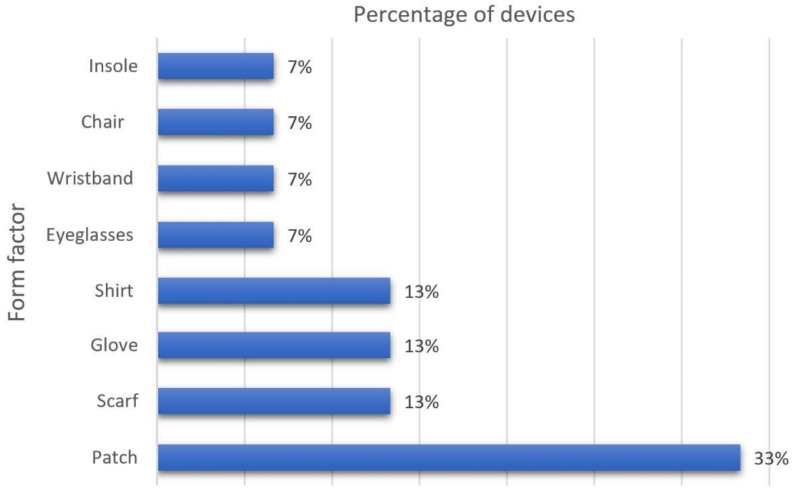
Classification of Non-Commercial Wearables by Form Factor.

Non-invasive sensors are common in the monitoring of physiological parameters during educational activities. Further, physiological parameter monitoring is relevant to research efforts that seek to develop and implement appropriate techniques for identifying student engagement in the teaching–learning process. Figure 7 introduces a graphic representation of our classification of non-commercial wearables for engagement detection with respect to their form factor.

Regarding to the contribution of non-commercial devices to assist and increase student engagement during educational activities, patches can detect signals such as ST, GSR, EDA, and ECG whose application is related to monitor the variation of the electrical properties of the skin through sweat. Meanwhile, Scarf can detect HR and EDA signals related to skin conductance monitoring. Gloves can detect PPG, EMG, HR, EDA, and RR signals related to monitor muscle contraction and physiological excitation. Regarding the Shirts, they can detect HR, ST, and ECG signals applied for performance monitoring associated with comprehension.

Under this context, EDA signals such as skin conductance are associated with stress and distress. While, HR signals are associated with stress, sadness, calmness, happiness, and excitement. EMG, RR, and PPG signals are related to happiness, anger, fear, disgust, and sadness. Moreover, all the detected physiological signals can identify the level of engagement of the students.

Table 5 summarizes our findings on the real-time monitoring capability of non-commercial wearables for engagement detection. As can be observed, 87% of these wearables reported in the literature can transmit physiological data in real time to other external devices for processing. Conversely, merely 13% of the wearables lack such real-time monitoring capability.

Table 6 summarizes our findings with respect to the main physiological signals involved in engagement detection in the case of non-commercial wearables. The majority of the non-commercial wearables reported in the literature can monitor two or more signals simultaneously.

Our findings revealed that heart rate is a key parameter measured by non-commercial wearables to detect and monitor student engagement during educational activities. Other important parameters include skin temperature, skin conductance, and electrodermal activity. Similarly, most of the reviewed devices primarily aim at improving student engagement levels during educational activities and thus academic performance.

## 5. Discussion

### 5.1. Challenges and Trends of Wearables for Engagement Detection

Even though technological progress has paved the way for the application of sensing technologies in educational research, further efforts are still needed.

- Engagement detection proposals need to increase the number of physiological signals being monitored.
- Despite having the ability to record physiological data in real time, some wearable devices still lack mechanisms for analyzing and processing such data.
- It is important improve technical aspects of the wearables, such as battery performance and device intercommunication for data transfer.
- Engagement research is a notorious opportunity in educational research, since physiological data analysis and processing techniques can be more efficient than other techniques, such as surveys, even though they cannot always speed up findings.
- Technological trends point toward the design of non-invasive, comfortable wearable devices, and thus provide manufacturers with a great opportunity to explore the efficiency and suitability of new materials and device shapes. A clear example of this is how sensors have been innovatively incorporated into chair and insole designs. Such designs explore the suitability of measuring relatively uncommon parameters, such as pressure on some parts of the body.

The main trends for engagement detection can be classified according to the methods for detecting students’ engagement. These methods can be:

(1) Automatic: sensor data analysis, log-file analysis and computer vision techniques.
(2) Semi-automatic: engagement tracing.
(3) Manual: Observational check-list and self-reporting.

We believe the recent trends are focused in developing new computer vision techniques for detecting facial expressions, gesture, posture and eye movement. For example, for gesture and posture have been developed such as Muse S band [133], Everion [134], Zephyr BioHarness [135], Xiaomi Mijia [136]. Muse S band can monitor Heart rate, EEG, PPG, posture, sleep level, Respiration Rate and Relax indicator. Everion device can identify Activity (move) indicator, Electrodermal activity/galvanic skin response, Heart rate, Respiration Rate, Sleep indicator, Relax indicator and Blood Oxygenation (SpO2). Chest Strap Zephyr BioHarness can measure Heart rate, body posture, activity intensity and SpO2. Xiaomi Mijia can monitor Heart rate, ECG, movement, Respiration Rate.

For facial expression and eye movement, smart glasses have been developed such as Oculus Quest Pro, iMotions Eye Tracking Glasses, Google Glass, Apple Glass and Tobii Pro Glasses 2, and eye tracking devices such as EyeTribe [137]. Oculus Quest Pro can be used to eye and face-tracking. iMotions Eye Tracking Glasses can be used to Track eye position, Facial Expression Analysis and movement to access visual attention in real time. Google Glass uses motion and voice recognition to process commands from the wearer and also operate the device with eye movements. Apple Glass can identify gestures and facilitate controls with eye movements. Tobii Pro Glasses 2 can be used to analyze human behavior in real time using eye tracking. EyeTribe Tracker allows controlling applications with user’s sight on desktop and tablet computers.

Additionally, with regards to sensor data analysis, embedded machine learning is used for interpreting data in Internet-of-Things applications. In this context, machine learning sensing capabilities are encapsulated in separate hardware components outside the central embedded processor and application code. Machine Learning (ML) is a subcategory of the Artificial Intelligence that refers to the process by which computers develop pattern recognition, or the ability to continuously learn and make predictions based on data, then make adjustments without being specifically programmed to do it.

### 5.2. Emerging Solutions

As wearable device technology progresses, solutions emerge to address common problems, such as battery performance, device size, and device shape. Graphene sensors are a clear example of innovative technological development. The use of graphene in wearable technology is an important contribution to the development of wearable devices for monitoring physiological, data such as brain signals. The recording of frequency levels using platinum and iridium electrodes is typically above 0.1 Hz, whereas graphene-based sensors could record brain signals below 0.1 Hz, thus increasing the amount of data that can be processed and improving brain-related research and its application in medicine. The use of graphene also has a positive impact on the battery performance of devices such as smartphones, since graphene is highly conductive [138].

Finally, device portability, versatility, simplicity, and real-time monitoring capabilities are important opportunities for improvement in sensing technology and ML techniques. ML techniques can process large volumes of data, and their application in biosensors can improve the monitoring of vital signs involved in the diagnosis of cardiovascular diseases, such as arrhythmias and coronary syndromes. As a key advantage, biosensor technology is cloud-compatible, which facilitates sensor signal processing and data storage. Further, it is easy to monitor health outside a clinical setting. The advantages of using biosensors and ML techniques mostly contribute to transforming raw data into understandable information, which in turn improves the performance of biosensors currently used for health monitoring, disease diagnosis, treatment evaluation, and food safety [138].

### 5.3. Limitations

This research has five main limitations. First, our review did not include a comparative analysis of the efficacy and reliability of the reviewed wearables in educational contexts. Second, we did not analyze to what extent each wearable actually contributes to increased student engagement during educational activities. Similarly, this review did not examine mobile applications for physical sign monitoring, since the non-commercial wearables reported in the literature were rarely linked to a mobile application for processing the data. Additionally, we did not analyze the usability aspects of the wearable devices or their user acceptance. Finally, our analysis of FDA approvals is partially incomplete, since most wearable manufacturers do not disclose such information publicly.

This review can be used by software engineers, developers, and computer scientists to develop mobile applications, educational platforms, or software to detect student engagement using physiological sensors and wearable devices. These systems can help improve the teaching–learning process to opportunely detect parameters such as frustration, boredom, stress, concentration, or distress that allow teachers to develop new learning strategies or improve existent.

## 6. Conclusions

This research is a review of current wearables used in educational environments for detecting and measuring student engagement in learning activities. Each device reported in the literature measures and analyzes different parameters, such as heart rate, skin temperature, EEG, ECG, respiratory rate, oxygen saturation, and blood pressure. Commercially available wearables for engagement detection are usually linked to a compatible mobile application to store and process physiological data in real time. In the educational domain, wearables and sensors for physiological signal monitoring can be used to identify the parameters that are key to student engagement detection during educational activities. The physiological signals being measured by a device strongly depend on the type of device. Wearable form factors such as smartwatches, chest patches, and wristbands are the most prominent in the market. As for non-commercial devices, the most cited form factors are patches (33%), shirts, gloves, and scarfs (13% of occurrence each).

The fact that wearables usually support real-time monitoring of physiological signals allows researchers to expand scenarios for data collection beyond the classroom and provides education experts with opportunities to redesign and propose meaningful teaching–learning methods and strategies. We consider our review of FDA approval status as a point of reference to judge both the reliability of the reviewed wearables in terms of data accuracy and their acceptance by users. In this sense, 33% of the wearables reported in the literature haven been approved by the FDA, 7% hold a Clear status, 10% have not been approved yet, and 51% maintain an unknown FDA status.

The scope of this research only covers the analysis of wearable devices currently available for student engagement detection during learning activities. Our review is not an analysis of the efficiency of such devices in terms of increased student engagement, nor is it a comparative analysis of the most suitable parameters for engagement detection. To conclude, we list relevant findings of this review:

- In total, 32% of the commercial wearable devices reviewed are smartwatches.
- In total, 40% of the commercial wearables have either an approved FDA status or a clear status.
- Engagement detection wearables commonly assess student physiological signals such as stress and surprise through physiological signals.
- Heart rate stands as the most prominent physiological signal measured by commercial devices.
- Patches are the most common form factor of non-commercial wearables for engagement detection.
- In total, 73% of the non-commercial devices reported in the literature support real-time physiological signal monitoring.
- Physiological signals commonly recorded by non-commercial devices are related to heart rate, skin temperature, skin conductance, EDA, respiratory rate, pulse wave, and oxygen saturation.

## Figures and Tables

**Table 1:** Classification of Biosensors for Cognitive Engagement Detection.

Type of Wearable Biosensor	Description	Wearable Biosensor Technologies	Wearable Device	Applications
Mechanical (Accelerometers and motion sensors) [69,70].	Accelerometers and motion sensors require the integration of another wearable physiological monitoring device as well as some type of computer software interface equipped with specific algorithms for signal manipulation and analysis. They are especially valuable when combined with wireless heart rate and ECG monitoring.	Accelerometer with ECG necklace Accelerometer and wireless heart rate monitor Motion sensor algorithm	Leap Motion Smartwatch Armband Headband Chest strap	Tracking gait Motion sensing
Physiological [69,70].	Physiological sensors can be used for predicting obstructive sleep apnea and monitoring heart rate, oxygen saturation, heart rate variability, breathing rate, and oxygen saturation. Further, these sensors can measure stress levels and mental fatigue.	PPG ring sensor PPG biosensors with smartphones PPG ECG magnetic earring and wireless earpiece PPG biosensors with GSR	Ring Muse band S Armband Headband Smartwatch Wristband Abdominal patch Chest patch Chest strap Vest Abdominal respiration belt GSR Velcro electrodes	Concentration monitoring
Biochemical [69,70].	Biochemical sensors can be used for non-invasive sweat monitoring through epidermal tattoo potentiometric sodium sensors with wireless signal transduction. Further, they are used for one-point wireless ECG acquisition with flexible polydimethylsiloxane (PDMS) electrodes.	Epidermal tattoo potentiometric sodium sensor Flexible PDMS electrode Flexible thick-film glucose biosensor Hydrogel-based (PAAM) photonic sensor Textile based patch with optical detection system Knitted fabric biocloth		Finger and limb motion detection

**Table 2:** Commercial Wearable Devices for Engagement Detection.

Manufacturer	Model	Form Factor	Sensors Used	Parameters	Physiological Signs	API	FDA
Biovotion™ [71]	Everion	Armband	HR sensor, PPG sensor	ST, SpO2, RR, HR, HRV, Sleep	Stress, relaxation	Everion device	Approved
Abbott [72]	FreeStyle Libre	Semi-invasive	Continuous Glucose Monitoring (CGM) sensor	ST, BP	Distress	Ambrosia	Approved (2020)
Halo Sport [73]	Halo Sport 2.0	Headphones	Electro neurostimulator	Neuropriming		Halo Sport	Approved
Scosche™ [74]	Scosche Rhythm24	Armband	HR optical sensor	PPG, HR, HRV, cadence, step tracking, burned calories, distance, speed	Stress, postures, surprise	ScoscheSDK24 Framework	Not Approved
Equivital™ [75]	LifeMonitor	Chest belt	ECG biosensor, HR sensor, medical-grade thermometer, and tri-axis accelerometer	ST, SpO2, RR, HR, HRV, GSR	Stress, engagement	Equivital	Approved
Med/Wise [76]	Gluco Wise ^®^	Clip (thumb, forefinger or earlobe)	CGM radio wave Sensor	Continuous glucose monitor (CGM)	No specified	Gluco Wise ^®^	-
Biobeat™ [71]	Biobeat™ Chest patch	Chest patch, wrist monitor	PPG	ST, SpO2, RR, HR, HRV, BP, ECG	Stress, surprise	Biobeat	Approved (2019)
G-Tech Medical™ [77]	G-Tech Medical™	Chest patch	EMG	EMG	Surprise	G-Tech Medical	-
Health Care Originals™ [78]	ADAMM-RSM	Chest patch	Acoustic sensor, HR sensor, temperature sensor	HR, ST, RR, cough	Stress, surprise	Health Care Originals	-
iRhythm™ [79]	Zio patch	Chest patch	HR sensor	ECG	Surprise	iRhythm™	Clear (2021)
Preventice™ [80]	Bodyguardian Heart	Chest patch	Accelerometer, ECG sensor	ECG	Surprise	Preventice™	Clear (2012)
VitalConnect™ [81]	Vital Patch	Chest patch	Accelerometer, ECG sensor, thermistor	ECG, HR, HRV, RR, ST, body posture, activity, BP, SpO2	Stress, surprise	VitalConnect™ website	Cleared
BioTelemetry™ [82]	BioTelemetry™	Chest patch	ECG sensor	ECG	Surprise	BioTelemetry™	Cleared
Kenzen™ [83]	Kenzen™	Chest patch	HR biosensor	Sweat, HR, ST	Stress, surprise	Kenzen™	No Approved
Theranica Nerivio Migra™ [84]	Theranica Nerivio Migra™	Chest patch	EMG sensor	EMG	Surprise	Theranica	Approved (2019)
Medtronic™ [85]	Zephyr BioHarness	Chest Strap	CGM sensor	HR, HRV, RR, body posture, activity intensity, acceleration, accelerometry, ST, burned calories, speed, distance, elevation, BP, SpO2	Stress, surprise	Zephyr: Developer and User Tools	Approved (2012)
Beddit™ [71]	Beddit Sleep Monitor	Contactless in-bed sensor	PPG sensor	RR, HR, sleep measures	Stress, relaxation	Beddit™	-
Beurer™ [71]	Beurer SE80	Contactless In-bed sensor	Respiratory rate sensor, HR sensor	RR, HR, sleep measures	Stress, relaxation	Beurer™	-
Cosinuss™ [71]	Cosinuss Two	Earbud	HR sensor, body temperature sensor, 3D accelerometer	HR, HRV, SpO2, activity	Stress	Cosinuss™	-
Yono™ [70]	Earbud	Earbud	Thermometer	ST	Stress	Yono™	-
BioIntellisense™ [71]	BioIntellisense Epidermal patch	Epidermal patch	HR sensor	ST, RR, HR, coughing, sneezing	Stress	BioIntellisense™	Approved (2019)
Bose^®^ [86]	SoundSport ^®^ Pulse	Wireless headphones	HR sensor	HR, PPG	Stress	Bose^®^ Connect	-
VivaLNK™ [87]	Fever Scout	Epidermal patch	ECG and HR sensors	ST	Stress, relaxation, surprise	VivaLINK	Approved (2017)
	Vital Scout	Epidermal patch	ECG and HR sensors	HR, HRV, RR, activity, sleep, stress levels	Stress, relaxation	VivaLINK	Approved (2019)
Spire Health™ [88]	Spire Health Tag	Fitness Tracker	HR, ECG, and RR sensors	HR, RR, breathing pattern, activity	Stress, relaxation, surprise	Spire Health™	Not Approved
Muse™ [89]	Muse S	Headband	EEG sensor	EEG, PPG, SpO2, breathing pattern, sleep tracking	Relaxation, concentration, postures, surprise, frustration, interest, laugh	Muse Developers	-
Motiv™ [90]	Motiv Ring	Ring	Accelerometer and PPG and HR sensor	PPG, HR	Stress, surprise	Motiv™	-
Oura™ [91]	Oura Ring	Ring	Body temperature sensor, optical, infrared sensors, 3D accelerometer and gyroscope sensor	PPG, HR, HRV, ST, RR, activity, sleep	Stress, surprise, relaxation	Oura Cloud API	-
Komodo Technologies™ [92]	AIO smart sleeve	Sleeve	ECG sensor	ECG, HR, HRV, activity intensity, SpO2, step tracking, distance	Stress, postures, surprise, interest, laugh	AIO Sleeve App	No Approved
Kinsa™ [71]	Kinsa	Smart thermometer	ST sensor	ST	Stress	Kinsa™	Approved (2013)
Orpyx™ [70]	Surro Gait Rx	Smartwatch, shoe insert, shoe pod	Pressure sensor	BP	Stress, surprise	Orpyx™	-
	Surro Sense Rx	Watch, shoe insert, shoe pod	Pressure sensor	BP	Stress, surprise	Orpyx	Cleared
Apple™ [93]	Watch Series 3,4,5	Smartwatch	Oximeter, electrical HR sensor, optical HR sensor, accelerometer, gyroscope sensor	Fitness and activity-tracking, ECG, PPG, HR, HRV, sleep quality, stress levels, RR	Stress, relaxation, postures, surprise, laugh, interest	Apple™ Developer	Approved
Empatica™ [94]	Embrace 2	Smartwatch	EDA sensor, peripheral temperature sensor, 3-axis accelerometer, gyroscope sensor	HR, HRV, EDA, ST, activity	Stress, engagement, laugh	Empatica™ for Developers	Approved (2018)
	E4	Bracelet	PPG sensor, 3-axis accelerometer, EDA sensor (GSR Sensor), infrared thermopile sensor	BVP, GSR, SC, HR, HRV	Stress, relaxation, arousal, excitement	Empatica™ for Developers	Not Approved
Fitbit™ [95]	Charge 4	Smartwatch	3-axis accelerometer, optical HR monitor, altimeter	PPG, HR, SpO2, activity, sleep	Stress, surprise, relaxation	Fitbit™	Not Approved
	Ionic	Smartwatch	3-axis accelerometer 3-axis gyroscope sensor, optical HR monitor, altimeter, ambient light sensor, vibration motor	HR, SpO2, activity, sleep	Relaxation	Fitbit™	Approved
	Versa 2	Smartwatch	3-axis accelerometer, optical HR monitor, altimeter, ambient light sensor, relative SpO2 sensor, built-in microphone	HR, guided breathing, SpO2, step tracking, distance, stress level, sleep	Stress, relaxation, postures, surprise	Fitbit™	Approved
Gyenno [96]	Gyenno Spoon	Spoon	Accelerometer	No specified	Stress	Gyenno	-
Gl Logic [97]	AbStats	Abdominal device	Vibration sensor, acoustic sensor	A telemetry monitor		GI Logic	-
Garmin™ [98]	Fenix 5	Smartwatch	HR sensor	HR, SpO2, activity, sleep	Stress, relaxation	Garmin™ Connect Developer	-
	Forerunner 945	Smartwatch	HR sensor	HR, SpO2, RR, activity, sleep	Stress, relaxation	Garmin™ Connect Developer	-
	Venu	Smartwatch	Pulse oximeter, HR sensor	HR, SpO2, RR, activity, sleep	Stress, relaxation	Garmin™ Connect Developer	-
	Vivoactive 4	Smartwatch	Pulse oximeter, HR sensor	HR, SpO2, RR, activity, sleep	Stress, relaxation	Garmin™ Connect Developer	-
	Vivomove 3	Smartwatch	Pulse oximeter, HR sensor	Step tracking, sleep quality, HR, stress levels, body composition, SpO2, intensity minutes, details of physical activity, breathing frequency	Stress, relaxation, postures, surprise	Garmin™ Connect Developer	-
Holter [75]	Stat-On™	Portable sensor	ECG sensor	HR, HRV	Stress	No specified	-
Honor™ [99]	Honor Watch Magic 2	Smartwatch	Accelerometer, gyroscope, magnetometer, optical HR sensor, ambient light measurement, and barometer	HR, stress levels, sleep quality, distance, speed, SpO2	Stress, relaxation, surprise	Huawei™ Developers	-
Huawei™ [100]	Huawei Watch fit	Smartwatch	6-axis IMU sensor (accelerometer sensor, gyroscope sensor), Optical HR sensor, capacitive sensor	HR, SpO2, sleep quality, stress levels, step tracking, distance	Stress, relaxation, postures, surprise	Huawei™ Developers	-
	Band 6	Smart Watch	Accelerometer, three electrodes, ECG sensor, barometric altimeter	ECG, SpO2	Stress, relaxation, postures, surprise	Huawei™ Developers	-
Mobvoi™ [101]	TicWatch Pro 2020	Smartwatch	HR sensor	HR, step tracking	Stress, postures, surprise	Mobvoi™ Developers	-
LifeBeam [102]	LifeBeam diy kit	Helmet	Optical sensor	HR, blood flow, and oxygen saturation		LifeBeam	-
Kuaiwear Kuai [103]	KUAI-Sport Headphones	Headphones	HR sensor and accelerometer	HR	Stress	No specified	-
Omron™ [104]	Heart Guide	Smartwatch	Accelerometer, PPG HR and oscillometer	PPG, HR, BP, ECG, step tracking, distance	Stress, postures, surprise, laugh, interest	OMRON API for Developers	Approved (2019)
OnePulse™ [105]	OnePulse™	Smartwatch	ECG sensor	HR, activity, sleep patterns	Stress, relaxation, surprise	Not specified	Approved
Samsung™ [106]	Samsung™ Gear Sport	Smartwatch	Accelerometer, Gyro Sensor, Barometer, HR monitoring sensor	HR, step tracking, sleep quality	Stress, relaxation, postures, surprise	Samsung™ Developers	-
	Samsung™ Galaxy Watch Active 2	Smartwatch	Accelerometer, barometer, gyroscope sensor, HR sensor	HR, sleep quality, stress levels, BP, distance, step tracking	Stress, relaxation, postures, surprise	Samsung™ Developers	-
SmartMonitor™ [105]	SmartMonitor™	Smartwatch	Accelerometer	Detects repetitive shaking motions, HR, activity	Stress, surprise	SmartMonitor™	-
Verily Life Sciences™ [107]	Verily Study Watch	Smartwatch	CGM sensor	Wireless monitor for pulse, HR, ECG, ST	Stress, surprise, laugh, interest	Verily	Approved (2019)
Viatom Technology™ [105]	Viatom Checkme O2	Smartwatch	Oximeter, HR sensor	HR, ECG, SpO2, activity tracker, ST, sleep monitoring	Stress, relaxation, surprise, laugh, interest	Viatom	Approved
Withings™ [108]	Withings™ ScanWatch	Smartwatch	ECG, oximeter	ECG, HR, SpO2, step tracking, distance, sleep quality	Stress, relaxation, postures, surprise, laugh, interest	Withings™ Developer	Approved
	Move ECG	Analog watch	Heart rate sensor, 3-axis accelerometer, 3-axis gyroscope sensor	HR, ECG	Stress	Withings Developer	-
Xiaomi™ [109]	Huami Amazfit Health Band	Smartwatch	ECG sensor, pedometer	HR, movement tracking	Stress, postures, surprise	Mi Developer	Approved (2019)
	Mi Smart Band 5	Smartwatch	ECG sensor	HR, sleep quality, step tracking, stress level, BP	Stress, relaxation, postures, surprise	Mi Developer	Not Approved
Sensoria™ [110]	T-Shirt Short Sleeve + HRM	T-Shirt	HR monitor	HR, speed, distance, step tracking	Stress, postures, surprise	Sensoria™ Platform	-
Ambiotex™ [111]	Ambiotex Smart Shirt	T-Shirt	ECG and HR sensors	Stress level, ECG, HR, HRV, step tracking	Stress, postures, surprise, laugh, interest	Ambiotex™	-
Tempdrop™ [70]	Tempdrop™	Underarm armband	Thermometer	ST	Stress	Tempdrop™	-
Carré Technologies™ [112]	Hexoskin Smart Garments	Vest	ECG sensor	ECG, HR, HRV, RR, stress level, effort, fatigue, activity intensity, acceleration, step tracking, sleep quality, SpO2	Stress, relaxation, postures, surprise, laugh, interest	Hexoskin Developers	-
Nuubo™ [113]	Nuubo Wearable ECG	Vest	ECG sensor	ECG	Surprise, laugh, interest	Nuubo™ Wearable ECG	Approved
Zoll™ [114]	Lifevest	Vest	Temperature sensor	ECG	Surprise, laugh, interest	Lifevest	Approved (2018)
AliveCor™ [115]	Kardia Band	Wristband	Electrodes	ECG	Surprise, laugh, interest	AliveCor™	Approved (2019)
Ava Science™ [70]	Ava Wristband	Wristband	2-wavelength optical PPG sensor	EDA, PPG, HR, ST	Stress, surprise, engagement, laugh	Ava	Approved
Sentio Solutions™ [70]	Feel	Wristband	EDA, PPG HR, and skin temperature sensors	EDA, PPG, HR, ST	Stress, surprise, engagement, laugh	Feel	-
iHealth™ [116]	Wireless Blood Pressure Monitor	Wristband	Oscillometer	BP, HR	Stress, surprise	iHealth™	Approved
MOCACARE™ [117]	MOCACuff	Wristband	HR sensor	BP, HR, SpO2	Stress, surprise	Mocacare™	-
Wavelet™ [105]	Biostrap Wristband	Wristband	3-axis accelerometer 3-axis gyroscope sensor	HR, HRV, SpO2, RR, in-depth sleep tracking	Stress, relaxation, postures, surprise	Biostrap	-
WHOOP™ [71]	WHOOP Wristband	Wristband	HR sensor	RR, HR, HRV, EDA, sleep	Stress, engagement, laugh	WHOOP™	-

Abbreviations: SpO2: Oxygen saturation; BP: Blood pressure; HR: Heart rate; HRV: Heart rate variability; EEG: Electroencephalogram; ECG: Electrocardiogram; PPG: Photoplethysmography; EMG: Electromyography; EDA: Electrodermal Activity; GSR: Galvanic Skin Response; RR: Respiratory rate; ST: Skin temperature.

**Table 3:** Main Physiological Signals for Engagement Detection.

Physiological Signal	FDA-Approved Devices	Non-FDA Devices	Total
Distress	1	0	1
Stress	22	39	61
Relaxation	10	19	29
Sleep disorders	1	0	1
Postures	4	14	18
Surprise	23	25	48
Concentration	0	1	1
Frustration	0	1	1
Interest	11	6	12
Laugh	10	6	16
Emotional arousal	0	1	1
Excitement	0	1	1

**Table 5:** Non-Commercial Wearables for Engagement Detection with Real-Time Monitoring.

Real-Time Monitoring	No. of Devices	Percentage
Yes	13	87%
No	2	13%

**Table 6:** Physiological Parameters for Engagement Detection by Non-Commercial Wearables.

Target Parameter	No. of Devices	Percentage
Heart Rate	11	73%
Skin Temperature	5	33%
Skin Conductance	4	27%
Electrodermal activity	4	27%
Respiratory Rate	2	13%
Pulse Wave	1	7%
Oxygen Saturation	1	7%

## Data Availability

Not applicable.
